# Self-harm in pregnancy and the postnatal year: prevalence and risk factors

**DOI:** 10.1017/S0033291721004876

**Published:** 2023-05

**Authors:** Karyn Ayre, Xiaoqin Liu, Louise M. Howard, Rina Dutta, Trine Munk-Olsen

**Affiliations:** 1 Section of Women's Mental Health, Health Services and Population Research, Institute of Psychiatry, Psychology and Neuroscience, King's College London, UK; 2 South London and Maudsley NHS Foundation Trust, Bethlem Royal Hospital, Monks Orchard Road, Beckenham, Kent, London, UK; 3 National Centre for Register-based Research, Aarhus University, Aarhus, Denmark; 4 Academic Department of Psychological Medicine, Institute of Psychiatry, Psychology and Neuroscience, Kings College London, London, UK; 5 Department of Clinical Research, University of Southern Denmark, Odense, Denmark

**Keywords:** Self-harm, perinatal, pregnancy, postnatal, prevalence, risk factors

## Abstract

**Background:**

Self-harm in pregnancy or the year after birth (‘perinatal self-harm’) is clinically important, yet prevalence rates, temporal trends and risk factors are unclear.

**Methods:**

A cohort study of 679 881 mothers (1 172 191 pregnancies) was conducted using Danish population register data-linkage. Hospital treatment for self-harm during pregnancy and the postnatal period (12 months after live delivery) were primary outcomes. Prevalence rates 1997–2015, in women with and without psychiatric history, were calculated. Cox regression was used to identify risk factors.

**Results:**

Prevalence rates of self-harm were, in pregnancy, 32.2 (95% CI 28.9–35.4)/100 000 deliveries and, postnatally, 63.3 (95% CI 58.8–67.9)/100 000 deliveries. Prevalence rates of perinatal self-harm in women without a psychiatric history remained stable but declined among women with a psychiatric history. Risk factors for perinatal self-harm: younger age, non-Danish birth, prior self-harm, psychiatric history and parental psychiatric history. Additional risk factors for postnatal self-harm: multiparity and preterm birth. Of psychiatric conditions, personality disorder was most strongly associated with pregnancy self-harm (aHR 3.15, 95% CI 1.68–5.89); psychosis was most strongly associated with postnatal self-harm (aHR 6.36, 95% CI 4.30–9.41). For psychiatric disorders, aHRs were higher postnatally, particularly for psychotic and mood disorders.

**Conclusions:**

Perinatal self-harm is more common in women with pre-existing psychiatric history and declined between 1997 and 2015, although not among women without pre-existing history. Our results suggest it may be a consequence of adversity and psychopathology, so preventative intervention research should consider both social and psychological determinants among women with and without psychiatric history.

## Background

Self-harm during pregnancy and/or the first postnatal year (‘perinatal self-harm’) often precedes maternal suicide (Knight et al., 2015), which remains a leading cause of maternal death in high-income countries (Goldman-Mellor & Margerison, 2019; Knight et al., 2015, 2018; Metz et al., 2016; Thornton, Schmied, Dennis, Barnett, & Dahlen, 2013). In addition to being a potential marker of suicide risk, perinatal self-harm has also been associated with an increased risk of negative obstetric and infant outcomes (Czeizel, Tomcsik, & Timar, 1997; Flint, Larsen, Nielsen, Olsen, & Sørensen, 2002; Gandhi et al., 2006) highlighting its inherent clinical importance.

Repeated cross-sectional observational data from several high-income countries, including Denmark, suggest acts of self-harm among young women have increased markedly over recent years (Gardner et al., 2019; McManus et al., 2019; Reuter Morthorst, Soegaard, Nordentoft, & Erlangsen, 2016) but temporal trends in perinatal self-harm vary internationally. For example, population registry data from Taiwan suggest a reduction in postnatal self-harm between 2002 and 2012 (Weng, Chang, Yeh, Wang, & Chen, 2016), in contrast with population data from Israel that suggested the rate was stable between 2006 and 2015 (Glasser et al., 2018). The rate of pregnancy self-harm reportedly remained stable between 2006 and 2012 in the USA (Zhong et al., 2016). However, a recent large US general population registry study reported the prevalence of acts of self-harm in the year preceding or following birth doubled between 2012 and 2017, describing this as a potential public health crisis (Admon et al., 2021).

Reported risk factors for self-harm in pregnancy and the postnatal year include younger age, unmarried status, psychiatric history, multiparity and adverse obstetric outcomes (Ayre, Gordon, Dutta, Hodsoll, & Howard, 2019; Comtois, Schiff, & Grossman, 2008; Gandhi et al., 2006; Schiff & Grossman, 2006; Weng et al., 2016). For women known to have mental disorders, there is some evidence that perinatal self-harm may be more common (Ayre et al., 2019) and that risk factors may differ between pregnancy and the postnatal period (Gressier et al., 2017; Shigemi, Ishimaru, Matsui, Fushimi, & Yasunaga, 2020). The profile of psychiatric disorders associated with perinatal self-harm remains unclear, with particularly sparse evidence regarding personality disorder, despite its strong association with self-harm outside the perinatal period (Haw, Hawton, Houston, & Townsend, 2001).

In this study, we aimed to describe temporal trends in the prevalence rate of self-harm in pregnancy and the postnatal year among women delivering liveborn infants, providing separate estimates for women with and without pre-existing psychiatric history. We also aimed to identify demographic, psychiatric and obstetric risk factors for self-harm separately in pregnancy and the postnatal period (one year after delivery).

## Methods

### Study design

Population-based cohort study using Danish nationwide administrative data.

### Study population

We identified 1 182 511 pregnancies of 684 490 women between 1997 and 2015. We excluded 594 pregnancies where gestational age at birth was less than 22 weeks or over 45 weeks, as deliveries outside these parameters are unlikely to result in livebirths, and excluded 9650 (0.8%) pregnancies for whom gestational age information was missing. We further excluded 76 pregnancies in women who emigrated before the index delivery. Consequently, 679 881 women constituted the final study population, comprising 1 172 191 pregnancies.

### Data sources

All residents of Denmark have a unique personal identification number which allows accurate linkage between and within registers. We utilized linkage between the Danish Civil Registration System (CRS) (Schmidt, Pedersen, & Sørensen, 2014), the Danish Medical Birth Registry (Bliddal, Broe, Pottegård, Olsen, & Langhoff-Roos, 2018), the Danish National Patient Registry (Lynge, Sandegaard, & Rebolj, 2011), the Danish Psychiatric Central Research Register (Mors, Perto, & Mortensen, 2011) and the Danish National Prescription Registry (Kildemoes, Sørensen, & Hallas, 2011).

The CRS was established in 1968 and among other things includes information on date of birth, emigration and death. The Danish Medical Birth Registry was established in 1968, computerized since 1973, and comprises data on all livebirths (Bliddal et al., 2018). The Danish National Patient Registry includes data on medical hospital inpatient treatment since 1977, emergency room visit and outpatient treatment since 1995 (Lynge et al., 2011). The Danish Psychiatric Central Research Register includes information on inpatient treatment at psychiatric hospitals since 1969 and emergency room visit and outpatient treatment since 1995 (Mors et al., 2011). Diagnoses are recorded as International Classification of Diseases, 8th Revision (ICD-8) codes until 1993 and ICD-10 codes from 1994 onwards (World Health Organization, 1994). The Danish National Prescription Registry includes information on the Anatomical Therapeutic Chemical (ATC) classification codes and date of prescriptions dispensed in community pharmacies in Denmark since 1995 (Kildemoes et al., 2011).

### Primary outcome

The World Health Organization definition of self-harm was used: ‘an act with non-fatal outcome, in which an individual deliberately initiates a non-habitual behaviour that, without intervention from others, will cause self-harm, or deliberately ingests a substance in excess of the prescribed or generally recognised therapeutic dosage, and which is aimed at realising changes which the subject desired via the actual or expected physical consequences’ (Platt et al., 1992). The outcomes of interest were any recorded inpatient or outpatient contact for self-harm at either medical or psychiatric hospitals occurring during pregnancy or the postnatal year. Codes used to define self-harm are listed in online Supplementary material 1 and were derived from a validated coding algorithm (Gasse et al., 2018).

### Covariates

We considered two different sets of covariates for the investigation of risk factors for pregnancy self-harm and postnatal self-harm. The following covariates were considered in the analysis of pregnancy self-harm: age at delivery (⩽24, 25–34 or ⩾35 years), maternal country of birth (Denmark or outside of Denmark), parity (one, two or more) and calendar year of delivery (1997–1999, 2000–2004, 2005–2009 or 2010–2015). The following variables were dichotomized (yes/no): previous psychiatric disorder at any time before pregnancy, hospital contact for psychiatric disorders in the 2 years preceding pregnancy, hospital contact for self-harm any time prior to pregnancy and parental psychiatric history before pregnancy. We also included antidepressant prescription within 2 years of pregnancy as a proxy measure for mental disorders managed in primary care.

We defined previous psychiatric history as a hospital contact for psychiatric disorders (ICD-8 codes 290–315; ICD-10 codes F00–F99) or one prescription for psychotropic medication (ATC codes N05 and N06), retrieved from the Danish Psychiatric Central Research Register and the Danish National Prescription Register. We categorized women with previous psychiatric history into six hierarchical groups (one being the highest), as described in Box 1. This hierarchical system reflects the organization of the ICD-10 diagnostic system and accounts for individuals with more than one recorded diagnosis. For example, if a woman has diagnoses of both schizophrenia and depression, schizophrenia would be defined as her diagnosis, as this is higher in the hierarchy. We defined parental psychiatric history as either parent having a hospital contact for a psychiatric disorder (any diagnoses within the F-chapter in ICD-10), prior to the index pregnancy. Antidepressant prescription within 2 years before pregnancy was defined as ATC code N06A recorded within the Danish National Prescription Registry.

Box 1. Grouping of mental disorders

1. Hospital diagnosis of psychotic disorders (schizophrenia and related disorders)
  (ICD-8: 295.x9, 296.89, 297.x9, 298.29–298.99, 299.04, 299.05, 299.09, 301.83; ICD-10: F20–F29)
2. Hospital diagnosis of mood disorders
  (ICD-8: 296.x9, 298.09, 298.19, 300.49, 301.19 excluding 296.89; ICD-10: F30–F39)
3. Hospital diagnosis of neurotic, stress-related and somatoform disorders
  (ICD-8: 300.x9, 305.x9, 305.68, 307.99 excluding 300.49; ICD-10: F40–F48)
4. Hospital diagnosis of personality disorder
  (ICD-8: 300–301 excluding 301.83; ICD-10: F60–F61)
5. Hospital diagnosis of other psychiatric disorders
6. No hospital diagnosis of psychiatric disorders but treated with psychotropic medication

For the analysis of postnatal self-harm, we defined the covariates mentioned above and the following additional individual dichotomized obstetric variables (yes/no): preterm birth (gestational age<37 weeks), low birth weight (birth weight <2500 g) and caesarean section (ICD-10 codes O82 and O84.2).

### Statistical analysis

The prevalence rate of pregnancy self-harm was defined as the proportion of pregnancies every 2 years or 2013–2015 with one or more self-harm event occurring in the index pregnancy. We defined the prevalence rate of postnatal self-harm in the postnatal period similarly. We used generalized estimating equations with an identity link to estimate the adjusted 1-year risk difference (Hanley, Negassa, Edwardes, & Forrester, 2003), i.e. the difference in the prevalence of pregnancy self-harm or postnatal self-harm from one year to the next, and adjusted for covariates mentioned above. Separate models were generated to study risk factors for pregnancy self-harm and postnatal self-harm. To investigate the risk factors for pregnancy self-harm, we followed each woman from the first day of pregnancy until the first self-harm event in the pregnancy, or delivery date; whichever came first. To explore risk factors for postnatal self-harm, we followed each woman from the day of delivery until the first self-harm event, emigration, death, 365 days after that delivery or 31 December 2016; whichever came first. We estimated the hazard ratios (HRs) with 95% confidence intervals (CIs) to indicate the risk of self-harm using proportional Cox regression models. Proportionality was verified by visually inspecting ‘log-log’ plots. A woman could contribute information from more than one pregnancy to the analyses. To account for the dependence between pregnancies in the same woman, we used the Huber sandwich estimator for the correction of standard errors. Data were missing in 12.2% and 12.6% of women for one or more covariates in the study of pregnancy and postnatal self-harm, respectively, and we applied 20 imputations using the Markov Chain Monte Carlo technique for imputing missing values (Royston & White, 2011). Data processing was conducted in STATA version 15.0 (StataCorp, 2017).

### Ethics

The study was approved by the Danish Data Protection Agency. No informed consent is required for studies based solely on registers in accordance with local and national rules and legislation.

## Results

A total of 679 881 women (1 172 191 pregnancies) were included in our study cohort, constituting 8.9 × 10^5^ person-years at risk for studying pregnancy self-harm and 1.2 × 10^6^ person-years for postnatal self-harm. During follow-up, we identified 377 women with records of self-harm during pregnancy and 742 women in the postnatal period. The prevalence rate of self-harm was 32.2 (95% CI 28.9–35.4) per 100 000 liveborn deliveries during pregnancy and 63.3 (95% CI 58.8–67.9) per 100 000 liveborn deliveries in the postnatal period. The characteristics of the study population are described in Table 1.

**Table 1.** Characteristics of the study population

Factor	Pregnancy self-harm			Postnatal self-harm		
	*N*	Cases	Cases/100 000 deliveries	*N*	Cases	Cases/100 000 deliveries
Age at index delivery						
⩽24	153 184	178	116.2	153 184	274	178.9
25–34	807 378	166	20.6	807 378	374	46.3
⩾35	211 629	33	15.6	211 629	94	44.4
Country of birth						
Denmark	987 846	281	28.4	987 846	517	52.3
Outside of Denmark	184 345	96	52.1	184 345	225	122.1
Parity						
1	523 574	207	39.5	523 574	307	58.6
⩾2	648 617	170	26.2	648 617	435	67.1
Previous psychiatric history						
No	940 373	183	19.5	934 496	327	35.0
Yes	231 818	194	83.7	237 695	415	174.6
Psychotic disorders	5231	16	305.9	5418	36	664.5
Mood disorders	19 327	34	175.9	21 128	82	388.1
Anxiety disorder	32 741	51	155.8	34 519	127	367.9
Personality disorder	6734	18	267.3	6817	23	337.4
Other psychiatric disorders	12 535	17	135.6	12 631	22	174.2
Psychotropic medication treatment	155 250	58	37.4	157 182	125	79.5
Antidepressant prescription within 2 years						
No	1 106 566	277	25.0	1 115 596	561	50.3
Yes	65 625	100	152.4	56 595	181	319.8
Psychiatric disorder within 2 years						
No	1 149 890	285	24.8	1 150 619	589	51.2
Yes	22 301	92	412.5	21 572	153	709.3
History of pre-conception self-harm						
No	1 152 775	275	23.9	1 152 500	602	52.2
Yes	19 416	102	525.3	19 691	140	711.0
Parental psychiatric history						
No	874 404	214	24.5	876 066	390	44.5
Yes	154 917	95	61.3	158 560	208	131.2
Missing	142 870	68	47.6	137 565	144	104.7
Calendar year at delivery						
1997–1999	195 145	57	29.2	195 145	110	56.4
2000–2004	318 431	100	31.4	318 431	208	65.3
2005–2009	314 467	104	33.1	314 467	219	69.6
2010–2015	344 148	116	33.7	344 148	205	59.6
Caesarean section						
No	–	–	–	958 082	589	61.5
Yes	–	–	–	214 109	153	71.5
Preterm birth						
No	–	–	–	1 103 297	664	60.2
Yes	–	–	–	68 894	78	113.2
Low birth weight						
No	–	–	–	1 114 719	678	60.8
Yes	–	–	–	50 972	56	109.9
Missing	–	–	–	6500	8	123.1

### Previous psychiatric history and perinatal self-harm

Compared to women without, women with a psychiatric history had a substantially higher prevalence of self-harm in both pregnancy and the postnatal period: 83.7 (95% CI 71.9–95.5) *v.* 19.5 (95% CI 16.6–22.3) per 100 000 liveborn deliveries in pregnancy and 174.6 (95% CI 157.8–191.4) *v.* 35.0 (95% CI 31.2–38.8) per 100 000 liveborn deliveries in the postnatal period. The prevalence rate ratio for the effect of psychiatric history on pregnancy self-harm was 4.3; for postnatal self-harm it was 5.0.

### Temporal trends in perinatal self-harm

**Fig. 1.**
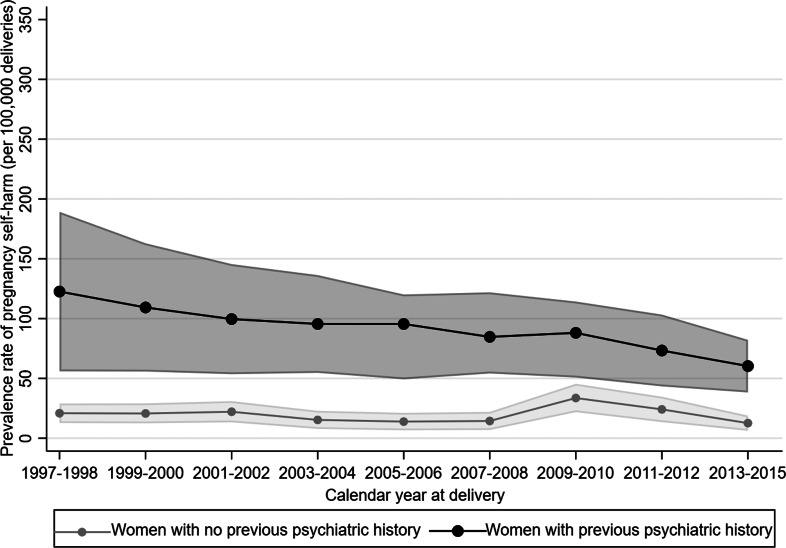
Prevalence rate of self-harm during pregnancy by previous psychiatric history in Denmark 1997–2015 (calendar year at delivery).

The temporal trend in the prevalence rate of pregnancy self-harm between 1997 and 2015 (calendar year at delivery), in women with and without previous psychiatric history, is shown in Fig. 1. Regarding women without a psychiatric history, the prevalence rate almost doubled around 2009–2010 but then slowly declined over subsequent years. For women with a psychiatric history, there was a gradual reduction over time. The adjusted 1-year prevalence difference in pregnancy self-harm was −0.4 (95% CI −0.9 to 0.1) per 100 000 liveborn deliveries for women without a psychiatric history and −9.8 (95% CI −16.0 to −3.7) per 100 000 liveborn deliveries for women with a psychiatric history.

**Fig. 2.**
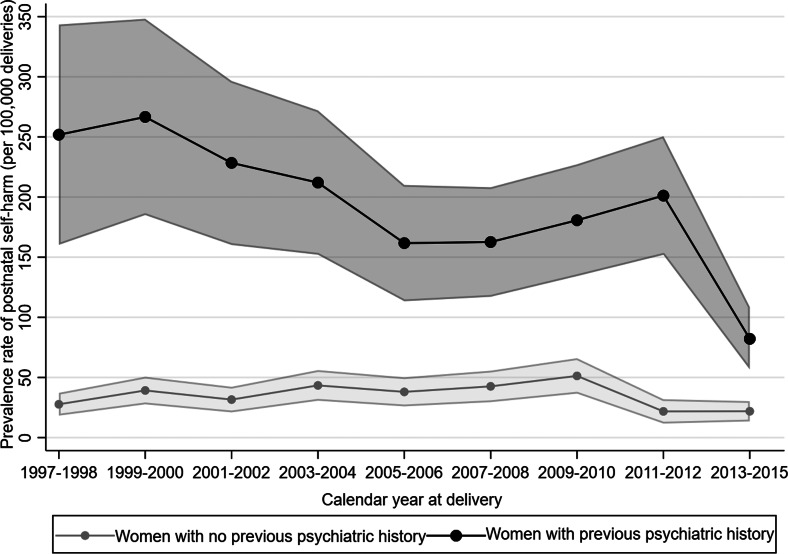
Prevalence rate of self-harm in the postnatal period by previous psychiatric history in Denmark 1997–2015 (calendar year at delivery).

The temporal trends in prevalence rates of postnatal self-harm between 1997 and 2015, in women with and without previous psychiatric history, are shown in Fig. 2. Regarding women without a psychiatric history, postnatal self-harm gradually increased, peaking in 2009–2010, then declined. For women with a psychiatric history, there was an overall reduction over time. The adjusted 1-year prevalence difference in postnatal self-harm was −0.6 (95% CI −1.4 to 0.1) per 100 000 liveborn deliveries for women without a psychiatric history and −34.5 (95% CI −42.8 to −26.2) per 100 000 liveborn deliveries for women with a psychiatric history.

### Risk factors for perinatal self-harm

**Fig. 3.**
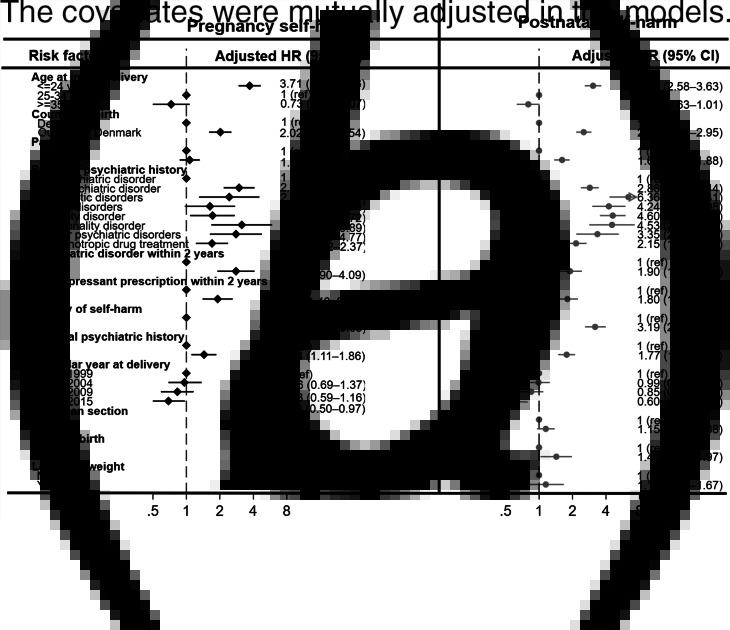
Adjusted hazard ratios of self-harm in pregnancy and postnatal period.

Adjusted hazard ratios for pregnancy and postnatal self-harm are presented in Figure 3. Risk factors common to both pregnancy and postnatal self-harm were younger age (⩽24 years), non-Danish birth, psychiatric history (ever and within 2 years), antidepressant prescription within 2 years, pre-conception self-harm and parental psychiatric history. Additionally, multiparity and pre-term birth were risk factors for postnatal self-harm.

There were however differences in the profile of psychiatric disorders associated with self-harm in pregnancy and the postnatal period (see Fig. 3). For pregnancy self-harm, women with personality disorder had the highest aHR (3.15, 95% CI 1.68–5.89). For postnatal self-harm, women with psychotic disorder had the highest aHR (6.36, 95% CI 4.30–9.41).

## Discussion

The prevalence rate of pregnancy self-harm was 32.2 (95% CI 28.9–35.4) per 100 000 liveborn deliveries and the prevalence rate of postnatal self-harm was 63.3 (95% CI 58.8–67.9) per 100 000 liveborn deliveries. To our knowledge, our study is the first to describe the prevalence trends of pregnancy and postnatal self-harm among women with and without psychiatric history. As expected, our results demonstrated that psychiatric history is a risk factor for perinatal self-harm. However, we have also shown that both lifetime and recent history increased perinatal self-harm risk. This is important, as it suggests that perinatal self-harm may not occur only in the context of new-onset perinatal mental disorder but rather can occur in the context of pre-existing illness. The importance of optimizing pre-conception mental health has recently been highlighted as a critical part of public health strategy to improve perinatal outcomes (Catalao, Mann, Wilson, & Howard, 2020), with increasing emphasis on the role of social determinants (Howard & Khalifeh, 2020; Spry et al., 2021).

Overall, we observed the prevalence rate of perinatal self-harm in women with a psychiatric history declined over an 18-year time period (1997–2015), which may be due to changes in mental health care both preconception and during pregnancy (e.g. alerting women with bipolar disorder of the high risk of postpartum psychosis). However, we did not observe an overall decline among women without prior psychiatric history as prevalence rates in this population remained largely unchanged, suggesting pre-conception health optimization strategies exclusively targeting women with pre-conception psychiatric history will not be sufficient to optimally reduce perinatal self-harm rates.

Our findings contrast with a recent US population study that suggested perinatal self-harm has increased between 2012 and 2017 (Admon et al., 2021), although direct comparison is challenging as the authors did not disaggregate pregnancy and postnatal self-harm. Moreover, data collection procedures and inclusion criteria for self-harm may also differ between Denmark and the USA. Admon hypothesized the observed increase could reflect increased detection, perhaps due to de-stigmatization of perinatal self-harm over time (Admon et al., 2021). However, a recent register-based study of self-harm among adolescents in the general population of Denmark found self-harm declined between 2007 and 2016 (Steeg et al., 2020) and we found younger age to be strongly associated with both pregnancy and postnatal self-harm in the survival analysis. One potential explanation could be that, in Denmark, younger mothers may be constituting a smaller proportion of the child-bearing population over time, perhaps due to more women deciding to delay having children until later in life. We speculate this is likely since the average age of women giving birth in Denmark has increased from 30.9 to 31.2 over the last10 years (Statistics Denmark, 2021).

We found self-harm was around three times more common in women under the age of 24 compared to other age groups. These findings are in line with existing studies of self-harm among women in the general population (McManus et al., 2016; Zubrick et al., 2016) and perinatal self-harm specifically (Comtois et al., 2008; Gandhi et al., 2006; Zhong et al., 2016). Proposed reasons for the increase in self-harm among young women include the disproportionate effects of economic disadvantage on this group (Agenda & the National Centre for Social Research, 2020). A recent cross-sectional investigation of pregnant women accessing routine maternity care in London (UK) concluded that women aged under 25, compared with women over 25, were at high risk of mental disorders (Lockwood Estrin et al., 2019).

We found risk factors for pregnancy and postnatal self-harm were broadly similar: age under 24 years, maternal birth outside of Denmark, psychiatric history, history of self-harm, antidepressant prescription within 2 years and parental psychiatric history. Postnatal self-harm was also associated with multiparity and preterm birth. There were subtle differences in the profile of psychiatric disorders associated with self-harm. Personality disorder was the most strongly associated disorder for pregnancy self-harm (aHR 3.15, 95% CI 1.68–5.89), whereas psychotic disorder was the most strongly associated for postnatal self-harm (aHR 6.36, 95% CI 4.3–9.41). There is very little research on women with personality disorder in the perinatal period (Howard et al., 2014) and our findings on self-harm during pregnancy highlights the importance of future research in this area. There has been more research into the increased risk of postpartum mood disorders including psychosis postpartum and our finding that mood disorder was associated with self-harm in the postnatal period (aHR 4.24, 95% CI 3.01–5.98) is therefore unsurprising. For all types of psychiatric disorder, the aHRs were higher postnatally than in pregnancy but this difference was particularly pronounced for psychotic and mood disorders.

Parental psychiatric history and prior history of self-harm were risk factors for self-harm in pregnancy as well as the postnatal period. An Australian general population cohort study found that pre-conception self-harm between the age of 20–29 years was associated with an increased risk of later perinatal depression and mother–infant bonding difficulties, compared with pre-conception self-harm as an adolescent (Borschmann et al., 2019). Notably, we found the adjusted hazard ratio for prior self-harm was markedly higher for pregnancy than postnatal self-harm: aHR 6.28 (95% CI 4.57–8.63) compared with 3.19 (95% CI 2.56–3.98) respectively. The reasons for this are unclear.

Women born outside of Denmark were around twice as likely to have self-harmed perinatally. Women born outside of Denmark have previously been found to have higher levels of psychiatric service contact during the perinatal period than Danish-born women (Munk-Olsen, Laursen, Mendelson, & Pedersen, 2010) so our finding could reflect higher detection rates. Alternatively, non-Danish-born women could be more likely to be marginalized and this could mediate self-harm, perhaps through social isolation (Calati et al., 2019). This population will include forced migrants, a population where pre and post-displacement stressors may contribute to an elevated risk of mental disorders, including perinatal disorders (Anderson, Hatch, Comacchio, & Howard, 2017; Jannesari, Hatch, & Oram, 2020).

Multiparity was a risk factor for self-harm in the postnatal period but not pregnancy. Possible mechanisms could be that having more children to care for along with a vulnerable newborn may constitute additional stress. We also identified preterm birth as a risk factor for postnatal self-harm. Babies born prematurely are at higher risk of short and long-term health complications (Vogel et al., 2018), so mothers may similarly be under more stress, either due to anxiety about, or from, managing these complications in the infant.

### Strengths and limitations

Our findings are based on routinely recorded and prospectively collected data, eliminating recall bias. The coverage of Danish registries is so comprehensive that the sample is population based and as a consequence selection bias is minimized (Thygesen & Ersbøll, 2014), and registry data allowed us to generate a very large sample size. To our knowledge, ours is the first general population study to identify detailed demographic, psychiatric, and obstetric risk factors for self-harm in both pregnancy and the postnatal period, allowing comparison of risk factor profile between the two periods. In addition, we add to the very sparse literature around trends in perinatal self-harm over time.

The main limitation is that we likely underestimate the true prevalence of perinatal self-harm as representative surveys from the UK suggest most people who self-harm do not present to hospital (McManus et al., 2016). There may be important risk factors for perinatal self-harm not readily available in population registries, including deprivation and domestic violence. The latter is known to be associated with suicidal ideation in the perinatal period (Gelaye, Kajeepeta, & Williams, 2016). We ascertained psychiatric disorders using ICD codes. The degree of granularity of each individual clinical assessment is unknown, including whether a structured clinical interview was used. This could affect the validity of personality disorder diagnoses, which are often complex (Pedersen & Simonsen, 2014). It is possible that clinician recording of self-harm changed over time, although we are not aware of any changes and this would not explain why self-harm declined among women with prior psychiatric history but not without. Finally, our strategy of cohort generation was to study pregnancies leading to live-born infants, meaning generalization of the findings to women experiencing stillbirth, miscarriage or termination of pregnancy is questionable.

## Conclusions

Both pregnancy and postnatal self-harm are more common in women with pre-existing psychiatric history. Younger, multiparous women with a history of prior self-harm and who experience pre-term birth are at particularly increased risk of perinatal self-harm, suggesting perinatal self-harm may be a consequence of adversity as well as psychopathology. Although we observed a decline in prevalence rate in women with pre-existing psychiatric history over an 18-year time period, this was not the case for women without a psychiatric history, suggesting pre-conception interventions to reduce perinatal self-harm should include women without pre-existing psychiatric history.

## Supplementary material

For supplementary material accompanying this paper visit https://doi.org/10.1017/S0033291721004876. click here to view supplementary material
