# Supplementary material for: Self-harm in pregnancy and the postnatal year: prevalence and risk factors
Source: Psychol Med. 2022 Jan 14;53(7):2895–903. doi: 10.1017/S0033291721004876 (PMC10235666; doi:10.1017/S0033291721004876)
Supplement: Supplementary file 1 [file S0033291721004876sup001.docx]

**Supplementary File 1: Self-Harm Codes**

*Nordic Medico Statistical Committee (NOMESCO):*

40 Attempted suicide; 41 Drug intoxication; 42 Self-mutilation; 48 Intentional self-harm, other specified; 49 Intentional self-harm, unspecified.

*International Classification of Disease (ICD)-8:* E950 - 959

*ICD-10:*

X60 – 84 Intentional self-harm

T39 Poisoning by nonopioid analgesics, antipyretics, and antirheumatics

T42 Poisoning by antiepileptics, sedative-hypnotic and anti-parkinsonism drugs

T43 Poisoning by psychotropic drugs, NOS

T58 Toxic effect of carbon monoxide

*Hospital contacts with the primary diagnosis as a psychiatric disorder (F chapter in ICD-10) and the secondary diagnosis as:*

*ICD-10:*

X60 – 84 Intentional self-harm

T36-50 Poisoning by drugs, medicaments, and biological substances

T52 Toxic effects of organic solvents

T53 Toxic effect of halogen derivatives of aliphatic and aromatic hydrocarbons

T54 Toxic effect of corrosive substances

T55 Toxic effect of soaps and detergents

T56 Toxic effect of metals

T57 Toxic effect of other inorganic substances

T58 Toxic effect of carbon monoxide

T59 Toxic effect of other gases, fumes, and vapors

T60 Toxic effect of pesticides

S51 Open wound of forearm

S55 Injury of blood vessels at forearm

S59 Other and unspecified injuries of the forearm

S61 Open wound of wrist and hand

S65 Injury of blood vessels at wrist and hand level

S69 Other and unspecified injuries of wrist and hand
